# AMULED: Addressing Moral Uncertainty using Large language models for Ethical Decision-making

**DOI:** 10.3389/frai.2026.1754973

**Published:** 2026-05-04

**Authors:** Rohit K. Dubey, Damian Dailisan, Sachit Mahajan

**Affiliations:** Computational Social Science, ETH Zürich, Zurich, Switzerland

**Keywords:** belief aggregation, ethical decision-making, large language models, moral uncertainty, reinforcement learning

## Abstract

**Introduction:**

We address moral uncertainty in reinforcement learning (RL) by proposing a framework that integrates multiple ethical theories into decision-making. Existing approaches rely on single moral frameworks or handcrafted rewards, limiting scalability and failing to capture moral pluralism. We introduce AMULED, a task-agnostic ethical layer that refines a pre-trained RL agent using large language models (LLMs) to provide multi-perspective moral feedback.

**Methods:**

Following initial training, the RL model is fine-tuned using LLM-generated feedback in place of human feedback. Five moral clusters—consequentialist, deontological, virtue, care, and social justice—assign belief values to candidate actions. These beliefs are aggregated using Belief Jensen–Shannon Divergence and Dempster–Shafer Theory to produce probability scores that serve as shaping rewards, while a KL-regularization term constrains deviation from the base policy. The framework is evaluated across two environments (Finding Milk and Driving and Rescuing), multiple LLM backbones, and alternative belief aggregation methods, with 50-run replicates.

**Results:**

AMULED improves ethical behavior without substantially degrading task performance. In Finding Milk, it increases desirable actions (63.1% more crying babies attended) and reduces undesirable actions (60.3% fewer sleeping babies disturbed), with only a 5.1% increase in path length. In Driving and Rescuing, it balances competing objectives more effectively than baselines, rescuing 38.4% more targets than human-feedback agents while maintaining lower collision rates and reduced policy degradation. Across experiments, BJSD-DST aggregation outperforms standard methods (e.g., voting, averaging) in handling conflicting moral signals and achieves the best overall performance on most metrics.

**Discussion:**

AMULED operationalizes moral pluralism through scalable, LLM-based feedback and provides a principled mechanism for resolving conflicting ethical signals. The framework demonstrates robustness across tasks and model variants, though performance depends on LLM reasoning quality and can degrade in spatially complex settings. These results suggest that LLM-driven belief aggregation offers a practical alternative to handcrafted rewards and human supervision for ethical decision-making in RL.

## 1 Introduction

Advances in artificial intelligence (AI) have led to significant developments of autonomous technologies. As these technologies make an ever-increasing number of decisions, these systems are increasingly scrutinized for ethical and safety issues (Chen, 2024). Autonomous vehicles, for example, must navigate complex moral dilemmas, such as choosing the lesser of two harms in potential accident scenarios. Although early research on endowment of AI agents with discernment (i.e., ensuring they act ethically and in line with human moral values) established the importance of ethical considerations in AI design (Allen et al., 2000; Wallach and Allen, 2008), these usually focused on singular moral theories such as deontological ethics (adherence to moral rules or duties) (Bringsjord et al., 2006) or utilitarianism (maximizing overall happiness or utility) (Russell et al., 2015). However, when creating ethically competent AI systems, a major challenge is widespread disagreement on the most appropriate ethical framework itself; there is always a *moral uncertainty* on which theory is correct within moral philosophy or across society (Ecoffet and Lehman, 2020). This diversity in moral philosophy complicates the design of AI agents that can be universally accepted as making morally sound decisions.

Recognizing the limitations of mono-theoretical approaches, recent research has explored the concept of moral pluralism and uncertainty in AI (Li and Du, 2017; Rhim et al., 2021). Moral uncertainty acknowledges that no single moral theory can be wholly applicable in all situations and therefore decisions must account for multiple ethical viewpoints (MacAskill et al., 2020; Ecoffet and Lehman, 2020). Efforts have been made to formalize moral uncertainty in computational models, allowing AI agents to integrate different moral considerations when making decisions (Lütge, 2019; Lindner and Bentzen, 2018). Reinforcement learning (RL), an AI framework in which agents learn and interact with their environment through trial and error, has been a key focus in efforts to integrate ethics into AI. Ethical actions can be learned by crafting appropriate rewards or penalties that effectively shape agent behavior to ethical outcomes (Abel et al., 2016; Hadfield-Menell et al., 2017), or by learning directly from human demonstrations through inverse RL (Ng and Russell, 2000; Ziebart et al., 2008).

Moral theories are inherently complex and often conflict with one another, making it challenging to translate abstract ethical philosophies into actionable frameworks for AI systems (Malle, 2016). Formalizing ethical principles without oversimplifying them requires exhaustive specification of rules (Dennis et al., 2016), which is unfeasible in complex, dynamic environments. In ethical AI, modeling rewards for ethical behavior is particularly difficult due to the vast and unpredictable nature of ethical dilemmas. Pre-coding rewards for all possible scenarios is unrealistic and limited by human foresight, as many dilemmas are context-dependent and difficult to anticipate. Therefore, this necessitates a more flexible and adaptive system that can respond to ethical challenges as they arise. While human oversight mitigates ethical risks in AI systems (Santoni de Sio and Van den Hoven, 2018), such data may be unavailable or often impractical for real-time decision-making tasks that require autonomy without human intervention (Cummings, 2014). Hence, gaps remain in the development of AI agents that can navigate ethical dilemmas and consider multiple moral frameworks. Existing approaches often fail to operationalize moral uncertainty effectively or translate the complexities of moral philosophies into practical algorithms for RL agents. Moreover, the dependency on human oversight is not scalable or feasible for many applications where AI agents must learn to act independently.

More recently, large language models (LLMs) have also pushed the frontiers of AI (Suri, 2024; Van Noorden and Perkel, 2023). These models have seen use beyond predicting the next token in a text sequence, with experiments showing their ability to drive agents that form complex social interactions (Park et al., 2023), collective decision making (Yang et al., 2024; Gudiño-Rosero et al., 2024), or other autonomous tasks (Wang et al., 2024). In a survey of open- and closed-source LLMs pitted against 1367 ambiguous and unambiguous moral scenarios, LLMs through their training on human data have encoded moral beliefs and would respond in a commonsense-manner (Scherrer et al., 2023). Although humans generally tend to be skeptical of AI decisions in ethical dilemmas, humans tend to agree with an LLM' assessment in moral scenarios (Garcia et al., 2024).

**Figure 1:**
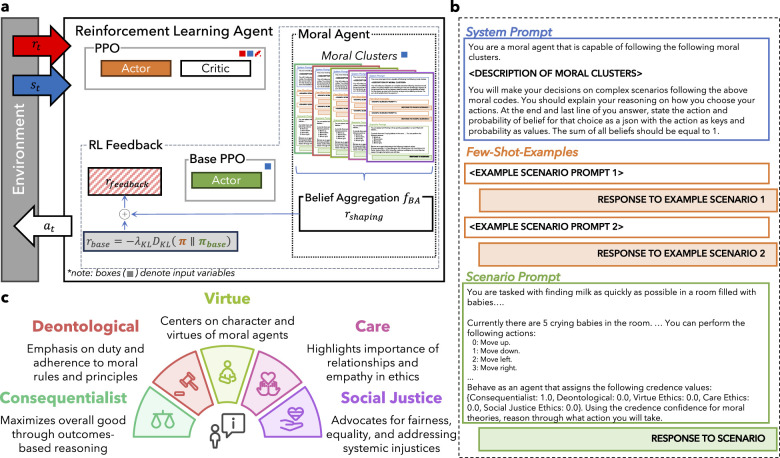
**(a)** Schematic diagram of the AMULED framework, which uses Reinforcement Learning with [AI] Feedback. The small colored boxes show which blocks have state *s* or reward *r* values as inputs. **(b)** LLM Prompt template used for the different Moral Clusters. **(c)** Different ethical frameworks used as moral clusters. Pseudo-code of the framework is detailed in Algorithm 1.

Thus, we introduce AMULED (Addressing Moral Uncertainty with Large Language Models for Ethical Decision-Making), a framework designed to incorporate insights from moral philosophy using LLMs into RL (Figure 1). AMULED translates utilitarianism, deontology, virtue ethics, and other moral philosophies into reward functions to enable agents to consider a diverse set of ethical principles (Sen, 2017). This approach recognizes the philosophical difficulty of comparing moral theories definitively and prioritizes the maintenance of diverse ethical considerations within AI decision-making.

Furthermore, our approach augments the lack of constant human oversight by empowering agents with the capacity to learn with autonomous ethical reasoning. Recent research has demonstrated that LLMs can develop reasoning capabilities that sometimes mirror human cognitive processes, suggesting their potential for ethical deliberation (Hagendorff et al., 2023). This is particularly relevant in high-dimensional, complex environments where data on ethical human feedback is impractical or unavailable. By generalizing the ethical training layer, we make it task-agnostic and applicable across diverse scenarios, introducing moral categories that consolidate multiple moral theories into practical algorithms. By mathematically modeling the conversion of moral theories into RL reward functions, we provide a practical solution for handling moral conflicts in uncertain scenarios (Gips, 2011). AMULED serves as a bridge to augment human feedback when missing or insufficient and thus it streamlines training and deployment, enabling faster, more scalable implementation while maintaining robust ethical guidance.

The practical implications of such a framework extend to various high-stakes domains where rigid rule-based systems often fail. For instance, in *geriatric care robotics*, an agent must frequently balance the patient's autonomy (e.g., a refusal to take medication) with the ethical duty of care (beneficence). Similarly, in *autonomous disaster response*, a drone fleet may face conflicts between utilitarian objectives (saving the most lives) and distributive justice (reaching isolated, vulnerable populations). AMULED provides the architecture to navigate these trade-offs by dynamically weighing these conflicting moral perspectives rather than defaulting to a pre-programmed heuristic.

Our work presents the following key contributions:

- Investigates moral uncertainty in complex, high-dimensional deep reinforcement learning environments.
- Proposes a generalizable ethical training layer applicable across diverse RL tasks.
- Develops a framework for categorizing and integrating multiple moral theories for nuanced decision-making.
- Translates philosophical insights on moral uncertainty into practical algorithms for AI systems.
- Establishes a mathematical model to map moral theories onto RL reward functions, addressing moral conflicts.

## 2 Related work

The study of AI ethics has attracted significant research attention across various domains, ranging from decision theory to reinforcement learning and large language models (Madanchian and Taherdoost, 2025; Laitinen and Sahlgren, 2021). In this section, we present an overview of key contributions in these areas, categorizing them into four sub-domains: Ethical Considerations in Decision Theory, LLMs and Their Role in Moral Reasoning, Reinforcement Learning in AI Ethics, and the Current State of the Art. The following works provide a foundation for understanding how ethical frameworks are integrated into AI systems and highlight both the challenges and progress made toward more responsible and ethical AI decision-making.

### 2.1 Ethical considerations in decision theory

Research in ethical decision theory for AI has explored both rule-based and learning-based approaches (Mahajan, 2025). Rule-based approaches often rely on moral philosophies such as deontology and utilitarianism. Deontological approaches emphasize adherence to moral rules or duties (Bringsjord et al., 2006), while utilitarian frameworks guide decisions based on outcomes that maximize overall good (Russell et al., 2015). However, these single-theory approaches struggle with complex moral dilemmas. To address this, researchers have proposed models incorporating moral pluralism and uncertainty, allowing AI to weigh different ethical perspectives (MacAskill et al., 2020; Lütge, 2019). Formalizations of Kantian ethics (Lindner and Bentzen, 2018) and multi-theory frameworks (Sen, 2017) illustrate attempts to integrate multiple moral theories into decision-making models.

### 2.2 LLMs and their role in moral reasoning

LLMs have become prominent in exploring moral reasoning capabilities. Several studies have assessed LLMs' ability to encode and apply moral beliefs through their training on human datasets. For instance, the Moral Turing Test (Garcia et al., 2024) found that humans often agree with LLM moral judgments. (Scherrer et al. 2023) demonstrated that LLMs encode commonsense moral beliefs. LLMs have also been used to simulate complex social interactions. (Park et al. 2023) created Simulacra, a society of LLM agents demonstrating emergent social behaviors. In collective decision-making tasks, Gudiño-Rosero et al. (2024) proposed LLM-Augmented Democracy, and (Yang et al. 2024) introduced LLM Voting for consensus building. These studies highlight LLMs' potential in moral and ethical reasoning tasks. However, despite their potential benefits, LLMs also pose significant risks and ethical challenges. For instance, biases inherent in training data can lead to unfair treatment of marginalized groups, perpetuating societal inequalities (Jiao et al., 2024). Additionally, the ability of LLMs to generate convincing yet false content raises concerns about misinformation and its impact on public trust and democratic processes (Coeckelbergh, 2025). The opaque nature of these models further complicates accountability, as determining responsibility for harmful outputs remains challenging (Jiao et al., 2024). Addressing these issues requires comprehensive strategies, including bias mitigation, transparency measures, and robust fact-checking frameworks (Xie et al., 2024).

### 2.3 Reinforcement learning in AI ethics

RL has been a core method for teaching AI ethical behavior. By shaping reward functions, researchers have guided agents to learn ethical outcomes (Abel et al., 2016; Hadfield-Menell et al., 2017). Inverse reinforcement learning (IRL) has been used to derive ethical policies from human demonstrations (Ng and Russell, 2000; Ziebart et al., 2008). Despite these advancements, RL faces challenges such as reward design complexity and the reconciliation of conflicting moral principles (Malle, 2016; Dennis et al., 2016).

Recent work also emphasizes human interaction and ethical constraints in applied RL settings. For example, Xiao et al. review human-AI interaction frameworks in reinforcement learning, highlighting trust, interpretability, and human-in-the-loop considerations, while Li et al. study the incorporation of explicit moral constraints into reinforcement-learning-based decision-making under partial observability (Xiao et al., 2025; Li et al., 2025).

Recent work explicitly addresses *safety* and operational guarantees in learning agents. The safe-RL literature studies constrained-MDP formulations and Lagrangian methods that enforce explicit safety constraints during training (Achiam et al., 2017), risk-sensitive and distributional objectives that mitigate catastrophic tail risks (Tamar et al., 2012; Chow et al., 2018), and safe exploration strategies such as shielding or reachability-based approaches that prevent unsafe actions during learning (Alshiekh et al., 2018; Berkenkamp et al., 2017). Control-theoretic frameworks using Lyapunov functions provide formal stability and safety certificates (Chow et al., 2018), while robust and offline RL methods aim to ensure safe behavior under uncertainty and limited data (Morimoto and Doya, 2005; García and Fernández, 2015). A comprehensive review of the literature can be found in (Gu et al. 2022).

### 2.4 State of the art

Several recent works address ethical AI decision-making. (Chen 2024) surveyed ethical challenges in autonomous decision-making, particularly in high-stakes domains like autonomous vehicles. (Duan et al. 2019) and (Mahajan 2024) discussed ethical framework implementations in AI systems. (Tolmeijer et al. 2020) reviewed the integration of moral theories into AI models. Additionally, LLM-driven approaches to AI ethics continue to gain traction, with studies such as (Suri 2024) and (Van Noorden and Perkel 2023) exploring LLMs' ability to navigate complex moral scenarios. These contributions collectively advance the field by integrating ethical reasoning into AI systems and highlighting gaps in moral uncertainty handling. Recent studies have also highlighted the challenges of integrating LLMs into ethical AI systems. For instance, biases in training data can lead to unfair outcomes, particularly for marginalized groups, as demonstrated by (Schramowski et al. 2022), who found that LLMs often encode human-like biases regarding moral judgments. Additionally, the opacity of LLMs complicates accountability, raising concerns about their use in high-stakes decision-making. Addressing these issues requires advancements in transparency measures and fairness constraints.

### 2.5 Summary of gaps and proposed innovations

Despite recent progress, current ethical RL frameworks typically rely on single moral theories or manual reward engineering, which fail to capture moral pluralism and are difficult to scale. Furthermore, existing methods for combining ethical perspectives often lack a rigorous mathematical basis for handling the uncertainty inherent in conflicting moral judgments.

To address these gaps, AMULED introduces three key innovations:

1. **Operationalizing moral pluralism:** we utilize LLMs to simulate five distinct moral clusters, treating them as independent “sensors” rather than relying on a single ethical viewpoint.
2. **Formal belief aggregation:** we apply Belief Jensen-Shannon Divergence and Dempster-Shafer Theory to mathematically resolve conflicts between these moral sensors, providing a robust measure of moral uncertainty.
3. **Scalable ethical feedback:** by replacing static rewards with dynamic AI feedback, we create a task-agnostic layer that guides agents through complex dilemmas without the bottleneck of continuous human supervision.

## 3 Methods

### 3.1 Development of moral clusters

The development of the moral clusters framework is grounded in a systematic analysis of ethical theories drawn from both classical and contemporary philosophical literature. Our objective is to construct a comprehensive yet practical structure that considers major ethical paradigms, serving as a foundation for implementing ethical reasoning in AI systems (Wallach and Allen, 2008; Anderson and Anderson, 2011).

We began with an extensive review of ethical theories, focusing on widely recognized works that span the spectrum of moral philosophy. This includes consequentialist, deontological, virtue-based, care-oriented, and justice-focused ethical frameworks (Powers, 2006). Key sources encompass seminal texts such as John Stuart Mill's *Utilitarianism*, Immanuel Kant's deontological writings (Alexander and Moore, 2024), Aristotle's *Nicomachean Ethics*, Carol Gilligan's work on care ethics (Gilligan, 2014), and John Rawls' *[A] Theory of Justice* (Pogge, 2007). This comprehensive review provides an overview of foundational principles and nuances within each ethical tradition (Dignum, 2018).

#### 3.1.1 Cluster identification

Drawing from the reviewed literature, we identified five primary clusters of ethical thought: Consequentialist Ethics, Deontological Ethics, Virtue Ethics, Care Ethics, and Social Justice Ethics (Figure 1c). These clusters were selected because they represent distinct approaches to ethical reasoning, encompass a broad spectrum of moral considerations, and are frequently discussed in both philosophical discourse and applied ethics (Tolmeijer et al., 2020). By organizing ethical theories into these clusters, we aim to capture the diversity of moral perspectives that could inform AI decision-making (Conitzer et al., 2017). This approach aligns with recent research highlighting the importance of comprehensive ethical frameworks in AI systems, particularly when dealing with decision-making dilemmas (Duan et al., 2019; Mahajan, 2024).

#### 3.1.2 Framework development

For each identified cluster, we developed a structured framework (details in Appendix S2) designed to balance philosophical depth with practical applicability for AI systems (Dennis et al., 2016). This framework includes a general description of the ethical approach, the key principles that unify theories within the cluster, representative ethical theories, key concepts inherent to each theory, and decision factors that could potentially be operationalized in an AI system. The selection of representative theories and concepts is based on their prominence in the literature and their potential for translation into computational models (Bench-Capon, 2020). In the Consequentialist Ethics cluster, for example, we focus on the principle of maximizing overall good, with utilitarianism serving as a representative theory (Abel et al., 2016). Key concepts such as utility, consequences, and the greatest happiness principle are emphasized, and decision factors involve assessing the outcomes of actions in terms of their utility contributions. Similar detailed frameworks were developed for the other clusters, ensuring that each ethical approach is thoroughly represented and that the essential elements could be mapped to computational considerations (Malle, 2016).

The initial framework has to undergo several iterations of refinement to enhance its coherence and applicability. We critically examine the internal consistency within each cluster and ensure clear distinctions between clusters to prevent conceptual overlap. This involves verifying that the selected theories adequately represent the diversity within each ethical approach and refining key concepts and decision factors to capture the essence of each theory while remaining amenable to quantification for AI applications.

### 3.2 Modeling morality as intrinsic reward: belief probability assignment

We draw inspiration from multi-sensor fusion literature (Chen and Deng, 2023), where measurements from different sensors can be converted into some plausibility estimate of what is defective in a system. The beliefs from a single sensor are termed Basic Belief Assignment (BBA), and combining the BBAs from multiple sensors yields a Basic Probability Assignment (BPA) (Zhao et al., 2022). Following this analogy, we envision the sensors as moral clusters, each with a set of probability estimates on which actions are best given the state. We can express this mathematically as the belief *B*_*i, j*_(*s*) ⇔ *B*_*i, j*_ of agent *i* on how good action *j* is, given the current state *s*. Thus, given *M* moral clusters and an environment with *A* actions, we can form a matrix of belief values. Without loss of generality, the moral clusters *M* can represent feedback from human agents, or as in the case of this work, LLM agents representing moral clusters. To make this belief matrix useful, we need a belief aggregation function *f*_*BA*_(**B**) → *r*_*j*_ that maps the belief values of agents into a reward *r*_*j*_ for each action *j*.

Multiple approaches can be used to serve as the aggregation function *f*_*BA*_(.). For example, *f* can be a majority-wins vote aggregation, where each agent “votes” on the action vote_*i*_ = argmax_*j*_*B*_*i, j*_, and the action with the most votes gets assigned *r* = 1. One could also use a maximum-belief approach by defining ${\tilde{B}}_{j}=\underset{i}{\max}B_{i,j}$, and setting $r_{j}={\tilde{B}}_{j}/\underset{i}{\sum }{\tilde{B}}_{i}$. Lastly, one can use a weighted average: $r_{j}=\underset{i}{\sum }w_{i}B_{i,j}/\underset{i}{\sum }w_{i}$, where *w*_*i*_ are weights of each agent. With *w*_*i*_ = 1, this weighted average reduces to the mean belief of an action. One expects these simple aggregation functions to work best when there is a strong agreement between agents on the probability values of each action. However, problems will arise when there are discrepancies between one or more agents. Thus, especially when ethics is involved, there is a need to treat such discrepancies with a more nuanced belief aggregation method.

In contrast to the above aggregation methods, (Xiao 2019) introduced a different aggregation method that makes use of a Belief Jensen-Shannon Divergence (BJSD) method to systematically measure the discrepancies and conflicts among BBAs, processes these measurements (details in Appendix S3), and finally employs Dempster–Shafer theory (DST) to arrive at the BPA (Dempster, 2008). This allows for a nuanced aggregation that accounts for the uncertainties inherent in human ethics, leading to more informed and comprehensive decision-making in moral dilemmas.

In multi-sensor data fusion, combining information from diverse sources is critical, yet challenging, particularly when addressing conflicting and uncertain data. Each sensor provides valuable insights into decision-making, but also introduces its own uncertainties. Similarly, different moral clusters, such as deontology, virtue ethics, and consequentialism, can be seen as “sensors” that guide ethical decision-making. These moral perspectives can conflict with one another and carry their own uncertainties. Treating these moral frameworks as sensors allows us to apply techniques from multi-sensor data fusion, particularly the BJSD and DST methods to effectively measure and moderate conflicts between evidence sources by incorporating both credibility and uncertainty metrics into the fusion process.

This approach begins by computing the BJS divergence. Let *A*_*j*_ be a hypothesis of the belief function *m*_*i*_: = [*B*_*i*,_*A*__1__, *B*_*i*,_*A*__2__, …*B*_*i*,_*A*__*j*__], and let *m*_*i*_ and *m*_*k*_ be two BBAs. The BJS divergence between two BBAs is given by


$$\begin{array}{l} BJS\left(m_{i},m_{k}\right)=H\left(\frac{m_{i}+m_{k}}{2}\right)-\frac{1}{2}H\left(m_{i}\right)-\frac{1}{2}H\left(m_{k}\right), \end{array}$$ (1)

where *H*(*x*) is the Shannon entropy. The BJS divergence quantifies the discrepancy and conflict between evidence clusters, which is then used to assign a credibility score for each evidence source, representing its reliability. Next, belief entropy is employed to account for uncertainties within each evidence cluster. This belief entropy captures the “volume of information” within each evidence source, offering a relative measure of each evidence's importance. By combining both the credibility degree and the belief entropy, Xiao's method dynamically adjusts the weight of each evidence cluster, minimizing the impact of conflicting information. These refined weights are then integrated using Dempster's combination rule, allowing for an adjusted belief assignment that yields more robust and interpretable fusion results.

Xiao's method is particularly suitable for combining human decision-making processes, especially in the context of moral dilemmas characterized by uncertainty. Human morality encompasses various frameworks (e.g., deontology, virtue ethics, and consequentialism), which reflect different values and principles. By using this method, we can incorporate multiple moral clusters, each representing different ethical perspectives. This approach supports a more nuanced and comprehensive understanding of moral decisions by mirroring the complexities of human decision-making. It enables us to account for conflicting beliefs and uncertainties inherent in human judgment, rather than relying on a single moral framework, and thus brings AI decision processes closer to how humans evaluate ethical situations in real-world contexts (MacAskill, 2014; Ugazio et al., 2021).

#### 3.2.1 Example scenario

To illustrate how this integration works in practice, consider a worked example in an autonomous vehicle scenario. The agent faces a dilemma: swerve left to avoid hitting a pedestrian (potentially endangering passengers) or continue straight (hitting the pedestrian but protecting passengers). The state (s) includes environmental details (e.g., road conditions, positions). Each of the *M* = 5 moral clusters (via LLMs) assigns belief scores *B*_*i, j*_(*s*) to actions (j) (e.g., swerve left, continue straight, brake hard) on a [0,1] scale.

- Consequentialist: High score (0.8) for swerve left if it maximizes overall utility (fewer total harms).
- Deontological: Low score (0.2) for swerve left if it violates a rule like “do not intentionally harm innocents.”
- Virtue: Moderate score (0.6) for brake hard if it reflects prudence and courage.
- Care: High score (0.9) for swerve left to prioritize vulnerable individuals.
- Social Justice: Moderate score (0.5) for continue straight if it avoids disproportionate harm to marginalized groups.

These form a belief matrix. BJSD then measures divergences (e.g., high conflict between deontological and consequentialist scores), and DST fuses them into BPAs, yielding dynamic rewards (e.g., swerve left: 0.7; continue straight: 0.3; brake hard: 0.6) discussed in Section 3.4.3. These rewards will then inform how the agent selects actions during learning, steering toward ethically balanced choices.

### 3.3 Large language models

We tested different state-of-the-art [large] language models as the backbone of the moral agents. Specifically, we chose the chat/instruct variants of GPT-4o-mini, Mistral nemo, and LLaMa-3.1 (8B and 70B variants), with GPT-4o-mini serving as our choice of LLM for AMULED, with the others serving as baselines. Setting the ‘temperature' parameter to 0 minimizes the variance in the models' responses and increases the replicability of our results. We structured the prompts (Figure 1b) into three main blocks: the system prompt, few-shot-examples, and the actual scenario prompt. The few-shot-examples were crafted to demonstrate the structure of a scenario prompt, and to incorporate techniques like chain-of-thought (Wei et al., 2022) to improve reasoning and model outputs.

### 3.4 Deep reinforcement learning

Deep Reinforcement Learning (Deep-RL) is a powerful paradigm for teaching agents to solve complex tasks by interacting with their environment. Specifically, it solves complex tasks that can be characterized as a Markov Decision Process (MDP) (Bellman, 1957), which is defined by the tuple 〈*S, A, R, P*〉. Here, *S* and *A* are sets of all possible states of the environment, and actions available to the agent, respectively. The reward function *R* determines the immediate reward obtained by the agent after taking an action in a given state, and *P* is the transition probability function that describes state transitions, given an action *a*.

Actions of an agent are selected using a policy π:S→*A*, and receives an instantaneous reward *r*_*t*_ = *R*(*s*_*t*_, *a*_*t*_), after which the environment transitions to a new state according to *P*. For control problems, one common objective is to find the policy π that maximizes the discounted expected return $G=\overset{T}{\underset{t=0}{\sum }}\gamma^{t}r_{t}$, where γ is a discount factor and *T* is a time horizon (Sutton and Barto, 2018).

#### 3.4.1 Reinforcement learning algorithm

Specifically, we use the Proximal Policy Optimization (PPO) (Schulman et al., 2017) as implemented in CleanRL (Huang et al., 2022), which uses neural networks to learn a policy π_ϕ_ and a value function *V*_θ_. The policy parameters ϕ is updated according to the equation


$$\begin{array}{l} \phi_{k+1}=\arg\underset{\phi }{\min}E_{t}\left(\frac{\pi_{\phi }\left(a|s\right)}{\pi_{\phi_{k}}\left(a|s\right)}A^{\pi_{\phi_{k}}}\left(s,a\right),\;g\left(\epsilon ,A^{\pi_{\phi_{k}}}\left(s,a\right)\right)\right), \end{array}$$ (2)

which uses common practices such as the *Generalized Advantage Estimation* *g*(ϵ, *A*) with advantage normalization and value clipping (Schulman et al., 2017). The weights of the value function neural network are updated according to


$$\begin{array}{l} \theta_{k+1}=\arg\underset{\theta }{\min}E_{t}{\left[V_{\theta }\left(s_{t}\right)-R_{t}\right]}^{2}. \end{array}$$ (3)

#### 3.4.2 Reward shaping

In general, we can express the rewards for a given task at a given timestep *t* as


$$\begin{array}{l} r_{t}=renv+c\cdot rshaping, \end{array}$$ (4)

where we break it down into two components: a *base reward*, which incentivizes the primary goal, and a *shaping reward*, which can address the secondary goals. Without shaping (i.e., *rshaping* = 0 base reward is typically the same as the instantaneous reward of the environment: *r*_*t*_ = *renv* = *R*(*s*_*t*_, *a*_*t*_). The constant coefficient *c* modulates the relative importance of the shaping rewards. Crafting a reward that fully satisfies the task and aligns with human values is not trivial (Butlin, 2021), particularly when multiple objectives must be satisfied (Korecki et al., 2024).

#### 3.4.3 Fine-tuning: reinforcement learning with [AI] feedback

We trained “base agents” solely using rewards that allowed them to complete their primary goals. This offers no guarantee of the agent's capabilities to also achieve the sub-goals. Instead of handcrafting the rewards to include incentives/penalties that steer the agent to learn the sub-goal, we instead employ reinforcement learning with [AI/human] feedback (RLHF) (Christiano et al., 2017; Lambert et al., 2022) to implicitly learn the desired behavior. However, instead of actual human feedback, we will use feedback from the LLM-moral clusters.

**Figure 2:**
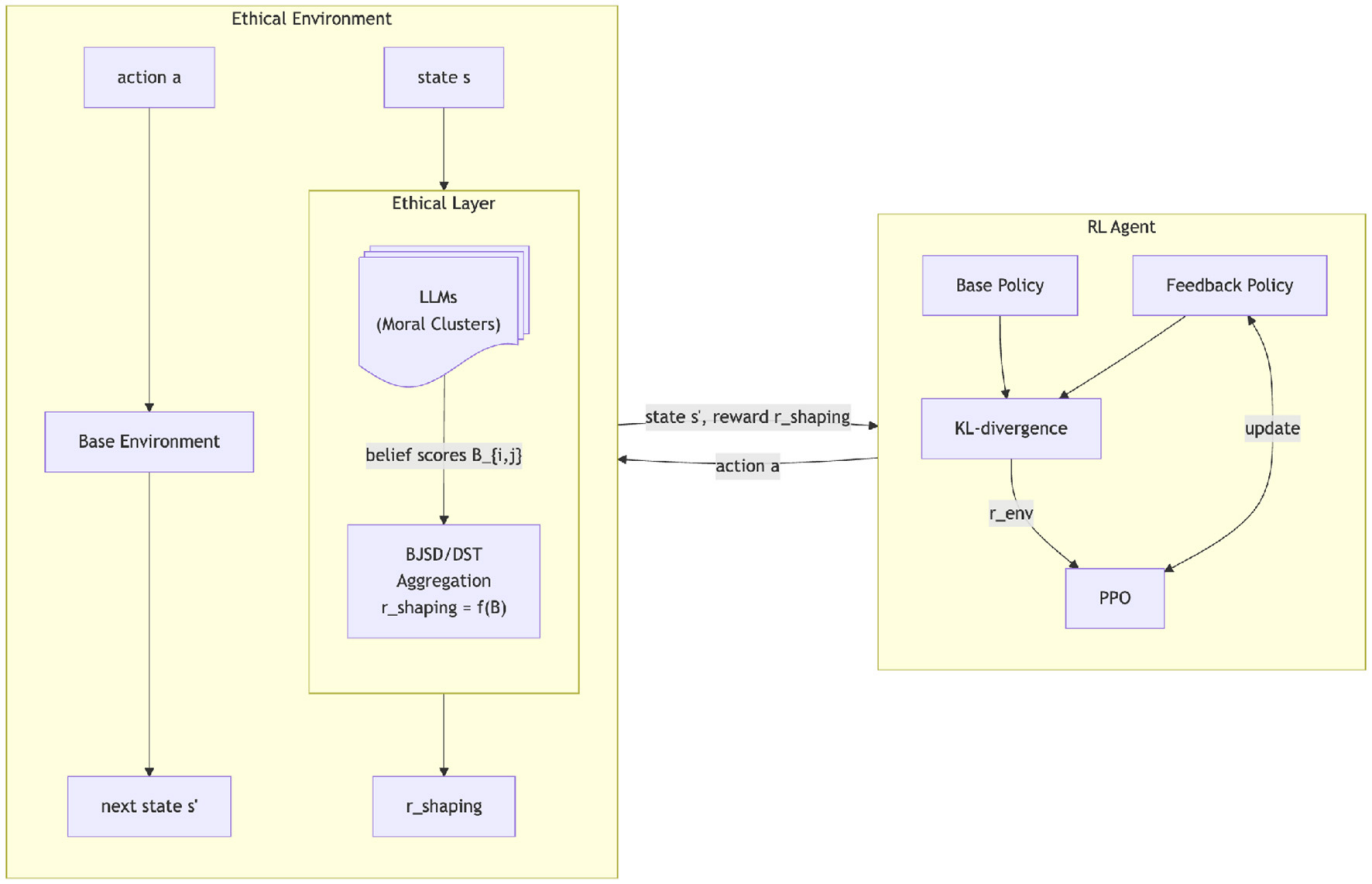
Fine-tuning loop with belief-to-reward flow. The RL agent interacts with the environment, with the ethical layer aggregating LLM beliefs into shaping rewards. The environmental reward from fine tuning is the KL-divergence of the base and feedback policies.

In fine tuning, we use the base learned policy π_*base*_ as a reference to train a copy of this base policy π using new rewards. The policy learned during fine tuning, denoted π_*feedback*_, should try to adopt the moral feedback from the moral clusters, without straying too far from the base policy that achieves the primary goal. We redefine the new rewards as *r*_*t*_: = *r*_*feedback*_ = *renv*+*rshaping*, with


$$\begin{array}{llll} renv & =-\lambda_{KL}D_{KL}\left(\pi feedback\left(a|s\right)||\pi base\left(a|s\right)\right), & rshaping & =f_{BA}\left(\text{B},a\right), \end{array}$$ (5)

where λ_*KL*_ controls how much deviations from the base policy are discouraged, and **B** is the belief matrix that gets translated into rewards for each action *a*_*i*_ in *A* (details in Appendix S3). Taking the Kullback–Leibler (KL)-divergence between the base and feedback policies quantifies the difference of the action-probability distributions (larger differences have higher *D*_*KL*_). Thus, using a negative factor for the KL-divergence discourages deviation from the base learned policy. The shaping rewards *rshaping* come from the aggregated belief values of the moral agents, where our proposed belief aggregation function *f*_*BA*_ uses BJSD and DST. Figure 2 illustrates this process in detail.

Algorithm 1 AMULED Framework. 
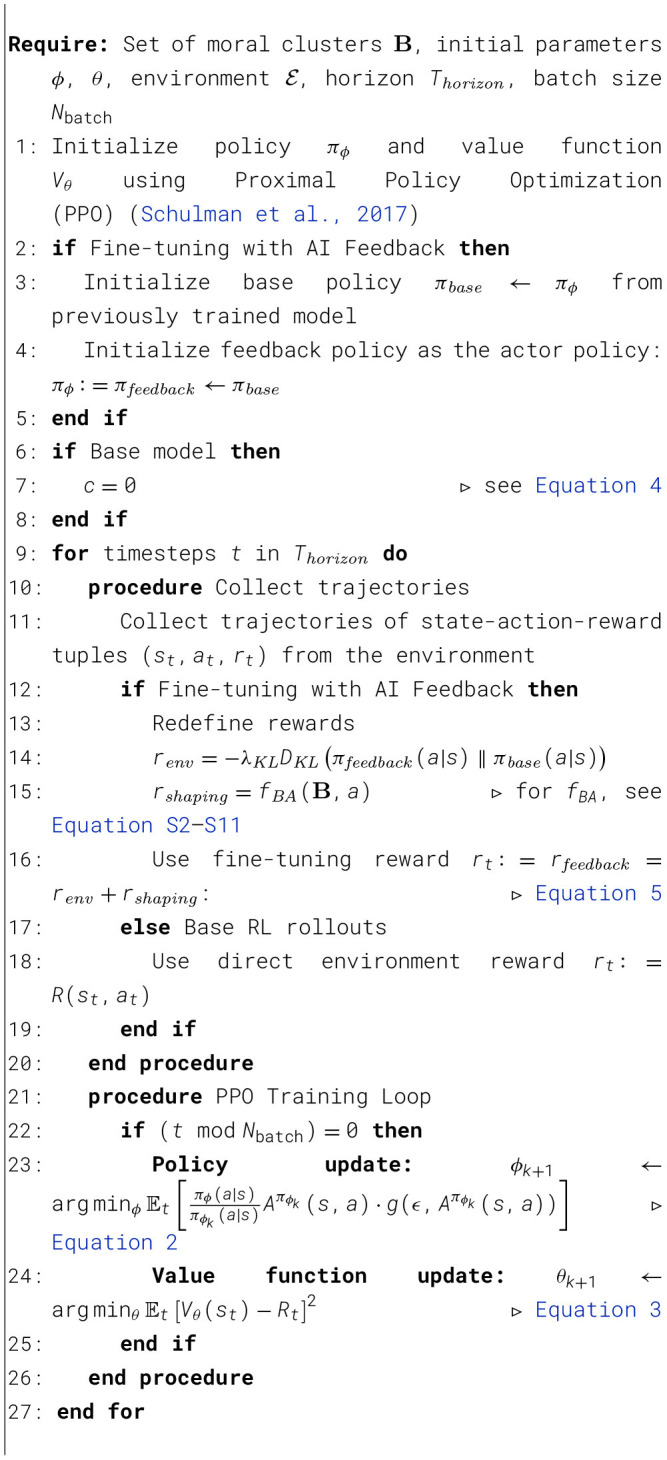

#### 3.4.4 Intuitive walkthrough of the fine-tuning process

Fine-tuning first requires a base policy π*base* that is trained on an environment without reward shaping. From this base policy, we initialize a new π*feedback* that is a copy of the π*base*. Now, we run the training algorithm once more following L2–5 and L12–16 of Algorithm 1. Now, the LLM-generated beliefs create a new reward following Equation 5 and act like a virtual ethical coach. During each episode, the pre-trained RL agent explores actions in the environment, and the ethical layer (via LLMs) evaluates them based on aggregated beliefs, producing shaping rewards that nudge the policy toward morally sound choices in place of the original environment rewards.

### 3.5 Experiment scenarios

#### 3.5.1 Finding milk

Route planning is a classic task for reinforcement learning and robotic techniques (Lin, 1992). In (Wu and Lin 2018), they created a modified Finding Milk scenario to use as a basic route planning problem considering ethical issues that should be carefully dealt with. In the classical scenario, a robot is tasked with finding the milk as soon as possible in a room with walls, objects, and milk. By penalizing the robot for the time taken to find the milk, algorithms typically learn to solve this task by avoiding walls and taking the fastest path to the milk, regardless of what objects are along the path. However, the modifications of (Wu and Lin 2018) introduced an ethical dilemma by changing the objects to crying or sleeping babies. Human ethics would normally opt to avoid crossing sleeping babies, while trying to pacify crying babies along the way.

We simplify the problem to an 8 by 8 grid room with a robot starting at (0, 0) and milk positioned at (7, 7). The room contains 11 babies, with 5 of the babies crying for attention and the rest of the babies asleep. For an agent aligned with human values, this task should be broken down as:

- **Primary goal**: Reach the milk in the least amount of steps possible;
- **Sub-goal**: pacify as many crying babies as possible;
- **Sub-goal**: avoid waking up sleeping babies.

In this MDP, the robot can choose from among four actions (up, down, left, right) that allow it to move to neighboring positions. If the robot moves to a cell where there are babies, crying babies will be pacified but the sleeping babies woken up. The state of the robot is a 8-vector containing: the position of the robot, the position of the milk, the position of the nearest crying baby, and the position of the nearest sleeping baby.

There are 147=3432 shortest paths to the milk, ideally with multiple paths that avoid all sleeping babies and pass through all crying babies.

#### 3.5.2 Driving and rescuing

Reinforcement learning has also seen widespread application in the design of autonomous vehicles. While autonomous cars paint an ideal picture where it can improve traffic efficiency and reduce traffic accidents, there remain ethical issues (Frank et al., 2019) concerning ethical decision-making that must not be overlooked. Our work uses a toy model presented by (Wu and Lin 2018), which is a simulation of car driving on 5 lanes. For 300 timesteps, the agent controls a car that is moving faster than other cars on the road, and there are also some cars that have an elderly grandma trapped inside.

For an agent aligned with human values, this task should be broken down as:

- **Primary goal**: Avoid collisions with other cars;
- **Sub-goal**: drive as steadily as possible (minimize lane changes);
- **Sub-goal**: rescue as many grandmas as possible.

For this task, the driver can choose to move in three ways (left, right, straight). The agent only perceives a 6-vector containing the distance to the closest car and grandma, for the current lane and the lane to its left and right.

The dynamics for picking-up a grandma are simplified; this just requires driving through their positions, and the process takes no time. Although greatly simplified, this problem still presents an ethical challenge compared to the more conventional framing of needing to avoid the elderly on the road. Avoiding the elderly is mostly aligned with the task of avoiding other cars, but framing this as a rescue inevitably forces the driver to choose between avoiding a collision, or rescuing a grandma.

### 3.6 AMULED framework

We integrate all of the previous methods in this section to create the AMULED framework (https://github.com/temetski/moral_agent). The AMULED framework develops a two-layered reinforcement learning (RL) model to balance primary and ethical objectives (see Algorithm 1). In the initial layer, the agent learns a policy for its primary task. Then, five distinct moral clusters evaluate each action's ethical appropriateness, assigning belief values based on the environment state. This state is encoded as a text prompt and processed with cluster-specific context to inform a shaping reward. The shaping reward, blended with the environment reward, adjusts the agent's actions toward ethical goals via reinforcement learning with feedback. In AMULED, we evaluate the effectiveness of the ethics shaping algorithm through four experiments, focusing on two key tasks (Section 3.5): (1) Finding Milk, where the agent performs a route planning task with additional ethical tasks, and (2) Driving and Rescuing, a more complex task involving a larger number of states that simulate realistic decision-making. In the following sections, we detail individual components of the AMULED framework.

### 3.7 Simulation studies

The core results of this paper come from the comparison between the base RL models, and the RL models trained with feedback (see Table 1 for the hyperparameters). The base RL models are trained using PPO for *T*_*horizon*_ steps (see Table 2 for complete hyperparameter settings), with the agent receiving the environmental rewards described in Equation 4 at each time step. This step produces two models: when *c* = 0, the **base** policy π*base* is trained solely on the primary goals; when *c* = 1, we get the **base + shaping** policy for handcrafted ethical reward functions.

**Table 1:** Comparison of metrics for the FindMilk scenario.

Model	Steps to milk	Crying babies passed	Sleeping babies passed
AMULED	14.2 (+5.1%) [ref.]	4.9 (+63.1%) [ref.]	0.6 (+60.3%) [ref.]
“Human” feedback	14.9 (ref.) [0.069]	3.0 (ref.) [ < 0.01]	1.5 (ref.) [ < 0.01]
Base	14.0 (+6.3%) [1.000]	4.0 (+34.9%) [ < 0.01]	3.8 (-163.0%) [ < 0.01]
Base + shaping	14.0 (+6.3%) [1.000]	5.0 (+67.1%) [1.000]	0.0 (+98.6%) [ < 0.01]
Consequentialist	14.0 (+6.3%) [1.000]	4.6 (+55.0%) [0.483]	0.9 (+37.0%) [0.594]
Deontologist	14.1 (+5.8%) [1.000]	4.0 (+32.9%) [ < 0.01]	1.2 (+20.5%) [ < 0.01]
Virtue	14.0 (+6.0%) [1.000]	4.6 (+54.4%) [0.445]	0.7 (+50.7%) [1.000]
Care	14.0 (+6.3%) [1.000]	5.0 (+67.1%) [1.000]	0.6 (+61.6%) [1.000]
Social justice	16.0 (-7.2%) [ < 0.01]	4.6 (+54.4%) [0.712]	0.7 (+52.1%) [1.000]
Moral	15.3 (-2.5%) [ < 0.01]	3.4 (+13.4%) [ < 0.01]	3.0 (-102.7%) [ < 0.01]
Majority vote	14.0 (+6.0%) [1.000]	4.7 (+56.4%) [1.000]	0.9 (+38.4%) [0.421]
Maximum belief	14.8 (+0.9%) [0.370]	4.9 (+63.1%) [1.000]	0.5 (+63.0%) [1.000]
Mean belief	14.0 (+6.3%) [1.000]	4.0 (+32.9%) [ < 0.01]	1.5 (-1.4%) [ < 0.01]
Mistral-nemo	14.3 (+4.4%) [1.000]	2.2 (-26.2%) [ < 0.01]	2.8 (-93.2%) [ < 0.01]
Llama3.1-8B	14.1 (+5.5%) [1.000]	4.0 (+32.9%) [ < 0.01]	1.1 (+26.0%) [ < 0.01]
Llama3.1-70B	20.0 (-33.6%) [ < 0.01]	4.8 (+61.1%) [1.000]	0.8 (+46.6%) [1.000]

In parentheses (), we highlight the relative differences of each method compared to “Human” feedback (+ percentages are improvements). In square brackets [], we show the *p*-values of a Mann-Whitney U-test with AMULED as the reference.

**Table 2:** PPO hyperparameters for base model training.

Hyperparameter	Value
Total timesteps	500,000
Learning rate	2.5 × 10^−4^
LR annealing	Linear
Discount factor γ	0.99
GAE λ	0.95
Parallel environments	4
Steps per rollout	128
Batch size	512
Mini-batch size	128
PPO epochs	4
Clipping coefficient ϵ	0.2
Value loss clipping	Yes
Entropy coefficient	0.01
Value function coefficient	0.5
Max gradient norm	0.5
Advantage normalization	Yes
Optimizer	Adam ($\epsilon_{\text{Adam}}=10^{-5}$)
Network architecture (actor & critic)	
Hidden layers	2 × 64
Activation function	tanh

We then take the **base** policy π*base* and use it to train a new policy π*feedback* for an additional *T*_*finetune*_ timesteps. The training loop still uses PPO, with the only difference being that we use the feedback rewards Equation 5. This step produces two models. The AMULED model uses the beliefs from the moral clusters *B*_*i, j*_, aggregated using the BJSD + DST belief aggregation function to produce the normalized BPA as rewards *rshaping*←*BPA*. As an alternative baseline, we use **“human” feedback** instead of an LLM to generate the belief probability values. Human trajectories are generated through stochastic policies that obey defined ethical rules, i.e, sets a higher belief probability actions that fulfill the sub-goals. The probability of such an action is set as the shaping reward: *rshaping*←*P*(*a*_*t*_|*s*_*t*_).

### 3.8 Ablation studies

In addition to the core results described above, we also performed three ablation studies to test the robustness of AMULED. Our first ablation study characterizes the different ethical values of each moral cluster (**consequentialist, deontologist, virtue, care, social justice**), compared to AMULED's aggregate approach. Here, we take the BBA of a single moral cluster *m*_*i*_ as the BPA. Additionally, we prompt the agent to act as a **moral** agent, without referencing the moral clusters to see the ethical biases of the LLM. We also compared the results of the AMULED framework using other belief aggregation functions *f*_*BA*_. Finally, we also compared AMULED, which uses GPT-4o-mini as its moral cluster, with other LLMs backends as moral clusters. To demonstrate the robustness of our results, each set of experiments is performed with 50 replicates, each run with a different random seed.

## 4 Results

We study two pertinent tasks: (1) Finding Milk and (2) Driving and Rescuing, which have been used in studying ethical decision-making frameworks (Wu and Lin, 2018). These tasks serve as proxies for real-world scenarios, encompassing a broader range of states and thereby demonstrating their applicability to everyday life situations. Fine-tuning the policy using feedback helps the RL agent incorporate ethical actions without deviating too much from the primary goal, effectively balancing ethics with operational efficiency.

### 4.1 Finding milk

**Figure 3:**
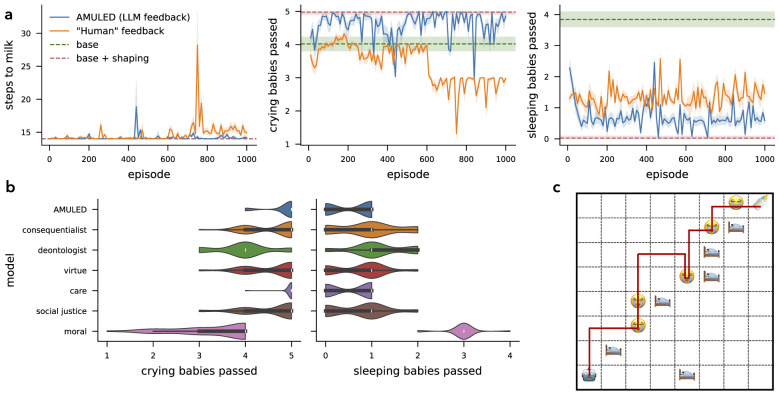
**(a)** Metrics evaluating performance of the agent on the primary goal (left-most) and sub-goals. AMULED learns the ethical task to near-perfection, although a hand-crafted shaping reward performs the best for this environment. Error bands reflect 95% confidence intervals of the mean. **(b)** AMULED is compared to the performance of agents prompted to act like pure moral clusters, and a “moral agent”. These values are measured from 50 episodes each. **(c)** Illustration of one of the trajectories learned by AMULED.

Figure 3a shows the learning curves of an agent trained with (moral or human) feedback. In the FindMilk environment (Figure 3c), the agent is tasked with reaching the location of the milk in the shortest possible time (primary task). However, the agent finds crying and sleeping babies on their way to the milk. The RL agents trained on the primary goal consistently learn to traverse the grid and find the milk in the shortest time possible. However, without informing an agent of the ethics of the problem, it will disregard any effects of meeting babies along its path. Introducing additional rewards *r*_*cry*_ = 1 and *r*_*sleep*_ = −1 for passing through babies would then help shape the agent's behavior to satisfy the moral sub-goals. This works really well for the FindMilk environment, but (as we will show in Section 4.2), in practice it is not trivial to assign the relative values of rewards for more complex tasks.

Table 3 shows the performance of AMULED in contrast with all of the other configurations. We find that fine-tuning the policy model (as an additional training layer) helps the agent incorporate ethical actions (63.1% more crying babies passed, with a 60.3% fewer at sleeping babies passed), without deviating too much from the primary task (5.1% less steps to milk). AMULED is the best performing model aside from the base+shaping approach, only because it is relatively easy to define a reward function that satisfies all objectives simultaneously. We also see that using the outputs of a belief model (combining the beliefs of 5 moral clusters) works even better than the synthetically generated human actions. However, for this environment, AMULED does not consistently find an ideal path to the milk. This is because the LLM (GPT-4o-mini), although it presents logical arguments for its actions on the basis of moral theories, sometimes fails in its spatial reasoning: for example, even if it has identified a sleeping baby to the right and it has explicitly identified that this goes against its moral goals, it will still go right because that brings it closer to the milk (even if going up also brings it closer to the milk, without passing through the baby). When we pass a similar prompt to GPT-4o (the best OpenAI model available at the time), the LLM is better able to reason out the spatial contexts.

**Table 3:** Comparison of metrics for the Driving scenario.

Model	Car collisions	Lane changes	Grandmas rescued
AMULED	2.0 (+51.7%) [ref.]	54.2 (-2.4%) [ref.]	13.6 (+38.4%) [ref.]
“Human” feedback	4.1 (ref.) [ < 0.01]	52.9 (ref.) [1.000]	9.8 (ref.) [ < 0.01]
Base	1.0 (+76.4%) [ < 0.01]	16.2 (+69.4%) [ < 0.01]	5.9 (-40.2%) [ < 0.01]
Base + shaping	12.5 (-207.9%) [ < 0.01]	167.1 (-215.9%) [ < 0.01]	19.1 (+94.9%) [ < 0.01]
Consequentialist	8.7 (-113.3%) [ < 0.01]	11.9 (+77.5%) [ < 0.01]	6.9 (-30.0%) [ < 0.01]
Deontologist	8.6 (-112.8%) [ < 0.01]	75.6 (-43.0%) [ < 0.01]	9.4 (-3.9%) [ < 0.01]
Virtue	4.1 (+0.0%) [ < 0.01]	74.5 (-41.0%) [ < 0.01]	14.7 (+50.0%) [1.000]
Care	3.5 (+12.8%) [ < 0.01]	60.0 (-13.4%) [0.210]	12.0 (+22.4%) [1.000]
Social justice	6.6 (-61.6%) [ < 0.01]	68.0 (-28.6%) [ < 0.01]	14.3 (+45.7%) [1.000]
Moral	7.4 (-81.8%) [ < 0.01]	53.8 (-1.8%) [1.000]	9.2 (-6.3%) [ < 0.01]
Majority vote	2.5 (+38.4%) [1.000]	42.5 (+19.6%) [ < 0.01]	13.5 (+37.6%) [1.000]
Maximum belief	2.7 (+33.0%) [0.677]	36.2 (+31.5%) [ < 0.01]	10.5 (+7.6%) [ < 0.01]
Mean belief	2.0 (+50.7%) [1.000]	33.6 (+36.4%) [ < 0.01]	12.2 (+24.5%) [1.000]
Mistral-nemo	4.3 (-5.9%) [ < 0.01]	67.7 (-28.0%) [ < 0.01]	13.1 (+34.1%) [1.000]
llama3.1-8B	16.1 (-296.6%) [ < 0.01]	130.3 (-146.5%) [ < 0.01]	13.7 (+40.0%) [1.000]
llama3.1-70B	2.0 (+50.2%) [1.000]	23.0 (+56.6%) [ < 0.01]	12.2 (+24.7%) [1.000]

In parentheses (), we highlight the relative differences of each method compared to “Human” feedback (+ percentages are improvements). In square brackets [], we show the *p*-values of a Mann-Whitney U-test with AMULED as the reference.

Because AMULED is a conglomeration of five moral clusters that guide the decisions of the agent, we can also gain insights on how it considers a diversity of moral philosophies by looking at how each individual moral cluster would guide the actions of the robot (Figure 3b). We note that AMULED has a statistically different behavior than social justice and moral clusters for “steps to milk”, deontologist and moral for both “crying babies passed” and “sleeping babies passed” (see *p*-values in Table 3). For the sub-goal of avoiding crying babies, we see that the “ideal” behavior is captured by the care moral cluster, which then more strongly aligns with the actions of AMULED. Another interesting thing to note is that, when we prompt the LLM with no explicit credence values (and thus just prompt it to behave as a “moral” agent), the agent passes through more sleeping babies and fewer crying babies. These are strikingly different results compared to how the moral clusters would act in this task.

### 4.2 Driving and rescuing

**Figure 4:**
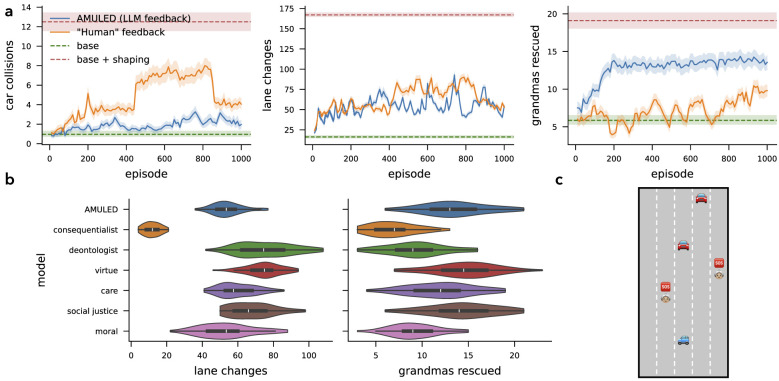
**(a)** Metrics evaluating the performance of the agent on the primary goal (left-most) and sub-goals. AMULED manages the tradeoffs between its conflicting goals much better than the other baselines. Error bands reflect 95% confidence intervals of the mean. **(b)** Comparison of AMULED with the performance of agents prompted to act like pure moral clusters, and a “moral agent”. These values are measured from 50 episodes each. **(c)** Illustration of the **Driving and Rescuing** environment.

In the Driving environment, we train an agent to simulate autonomous driving and avoid collisions with other cars on a five-lane road (Figure 4). Besides other cars, the environment also has some grandmas trapped in traffic. The agent will not factor “rescuing” grandmas (simplified as driving through the same lane as the grandma) in its decisions, unless given explicit rewards to shape its behavior. One way of defining the shaping reward is *r*_*shaping*_ = 400*r*_*grandma*_+20(lane_*t*_ = =lane_*t*−1_), where rescuing grandmas and staying on the lane are incentivized. This task presents a challenge to the agent, as avoiding car collisions can conflict with the secondary goals, depending on the stochasticity of the environment.

Table 3 shows the performance of AMULED in contrast with all of the other configurations. Here, the “Human” feedback approach produces a good baseline across all metrics, considering that lane changes is not as safety-critical as the other two metrics. Similar to the FindMilk scenario, we find that the base RL model can be trained to avoid collisions well. Using both LLM and human feedback, the agent incurs more car collisions than the base RL agent. On the other hand, we see here that defining the shaping reward can completely shift the agent behavior to prioritize rescuing grandmas (+94.9%), at the expense of excessive lane changes and collisions (-215.9% and -207.9%, respectively). Although the synthetic human actions were also much more inclined toward rescuing grandmas, we observe that the agent does not seem to rescue as many grandmas as the feedback samples (Figure 4a). The RLHF approach rescues around 10 grandmas on average while limiting the collisions to around 4. In comparison, AMULED performs quite well at balancing the secondary goals (rescuing grandmas and remaining in its lane) against the primary goal. Although AMULED does not save as many grandmas as the agent trained on hand-shaped rewards (-2.4%), it saves more grandmas (38.4%) than the agent trained on human feedback, while incurring a smaller forgetting cost (51.7% better).

When compared with the performance of agents acting as pure moral theories, we see more variance in the sub-goal performance of each moral cluster. AMULED has a statistically different behavior than all clusters across all goals, except for car and moral clusters for “lane changes” and virtue, care, and social justice for “grandmas rescued” (see *p*-values in Table 3). Most strikingly, the consequentialist approach favors “inaction”, which leads to staying more in lane and fewer rescues of grandmas (-30%) than the other approaches.

### 4.3 Sensitivity to λ_*KL*_

**Figure 5:**
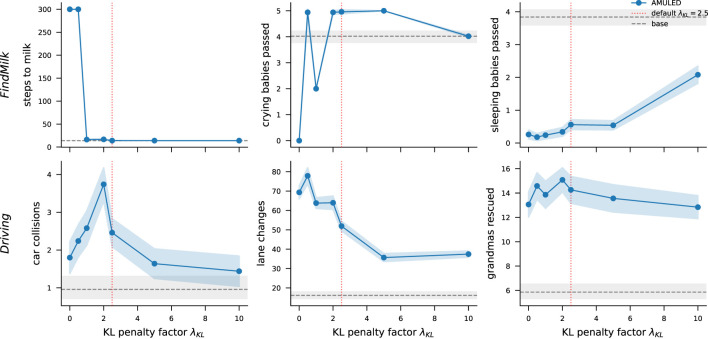
Sensitivity analysis of the λ_*KL*_ parameter. The value of λ_*KL*_ = 2.5 provides the best tradeoff between adhering to the original learned policy and deviations that allow for the completion of sub-goals.

We also performed a sensitivity analysis of the λ_*KL*_ parameter, which disincentivizes large deviations from the originally learned policy during finetuning. Specifically, we vary λ_*KL*_ across a range of values to study the trade-off between preserving the base policy and incorporating ethical feedback. Lower values allow greater policy updates driven by moral shaping rewards, potentially improving ethical alignment but risking instability in task performance, while higher values constrain updates, preserving task performance but limiting ethical adaptation. The results (Figure 5) show that intermediate values of λ_*KL*_ achieve a balanced trade-off, demonstrating the robustness of AMULED to reasonable variations in this parameter.

### 4.4 Aggregation of moral beliefs

Because we deal with a pluralistic moral framework, a key aspect of AMULED is an aggregation mechanism that combines beliefs from each moral cluster. Taking inspiration from multi-sensor data fusion, AMULED employs a belief aggregation system that combines the beliefs of the five moral clusters to a resultant vector of values corresponding to each action.

**Figure 6:**
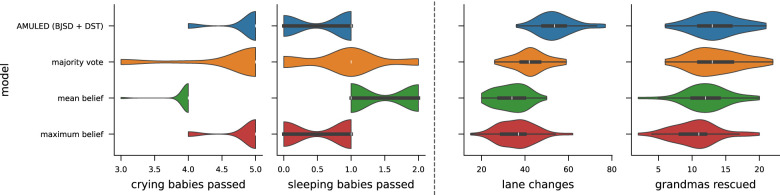
Comparison of AMULED with the performance of agents trained with alternative belief aggregation functions. The two left panels are for FindMilk, while the two right panels are for Driving. These values are measured from 50 episodes each.

For AMULED, we choose an aggregation method developed by (Xiao 2019) to generate the combined belief probability assignment (BPA) for each action. We take the BPA as the shaping rewards during learning with feedback. In principle, this is not the only way to aggregate the belief values of the different moral clusters. For comparison (Figure 6), we look at three other aggregation methods: (i) **majority vote**: each moral cluster “votes” for the action with the highest belief; then, the action with the most votes is assigned an aggregated belief value of 1; (ii) **maximum belief**: the aggregated belief value of an action is the highest belief value of all moral clusters; and (iii) **weighted average**: the belief value of an action is the weighted average of beliefs across all other moral clusters. For simplicity, we set the weights to be 1 (i.e., the weighted average is the mean).

For the FindMilk scenario, we see that AMULED and the max aggregation method have the most similar results for both sub-goals. However, only the mean belief aggregation method has statistically significant difference, and only for the sub-goals. On the other hand, AMULED is only similar to voting, and only for the “grandmas rescued” sub-goal. It has significantly different behavior for the lane changes sub-goal for all aggregation methods, and to maximum belief for grandmas rescued. Overall, AMULED mostly achieves the sub-goals best among the aggregation approaches, except for the “lane changes” sub-goal. Certainly, the choice of aggregation function can have statistically significant impacts on the outcomes for different types of environments.

Compared to traditional methods, BJSD–DST excels in handling uncertainty. For instance, in our experiments, we see very different outcomes that each moral cluster focuses on, which AMULED (BJSD–DST) can integrate to get overall good results. It produces the best overall outcome across all metrics for FindMilk (see Table 1), while being the best for car collisions and grandmas rescued in Driving (see Table 3) vs. majority vote, maximum belief, and minimum belief. However, traditional aggregation methods perform better on lane changes, which is the least consequential metric of the three. Traditional aggregation leads to suboptimal rewards in conflicted states (e.g., up to ~60% higher expected loss in some metrics), while BJSD-DST explicitly models conflicts for more interpretable fusions.

Intuitively, BJSD can be analogized to measuring ethical disagreements like sensor noise in a multi-sensor system: it quantifies how much the moral clusters “disagree” by calculating divergences between their belief distributions, highlighting conflicts that need resolution. DST then acts like negotiating a group consensus, fusing these beliefs by assigning credibility weights to reduce the influence of noisy or conflicting inputs, resulting in a balanced probability assignment that reflects collective ethical wisdom rather than a simple average.

### 4.5 Comparison with different LLMs

**Figure 7:**
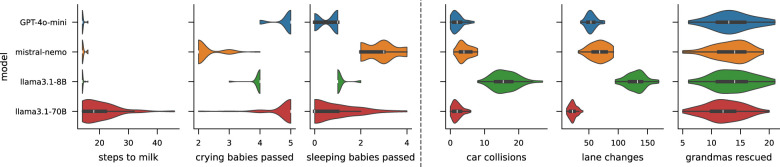
Performance of AMULED using different LLMs as the moral agents. The two left panels are for FindMilk, while the two right panels are for Driving. OpenAI's GPT-4o-mini performed the best among LLMs across the different metrics, which is why it was used to produce the results of the rest of the paper.

The results of AMULED presented so far were obtained using OpenAI's GPT-4o-mini. Because the language model served as a core element in guiding ethical decision making, we also compared the performance of AMULED that uses other language models (Figure 7). Although there is no official documentation on the exact size and architecture of GPT-4o-mini, some benchmarks put it on par with ~70 Billion parameter models (White et al., 2024). We compared it against Mistral NeMo (12B) and LLaMa 3.1 (8B and 70B). Overall, GPT-4o-mini performs the best for the primary and sub-goals, with statistically significant differences observed across both primary and subgoals, except for “grandmas rescued” in Driving. The bigger LLaMa 3.1 70B also performs well for the Driving scenario and has a good average performance on the sub-goals of FindMilk, however, it fails to consistently achieve the primary goal for the FindMilk scenario. This comparison suggests that GPT-4o-mini offers a distinct advantage in *instruction adherence*, a critical capability when maintaining the rigid philosophical personas required by the moral clusters. While larger models like LLaMa-3.1-70B possess strong general reasoning, our metrics indicate they struggled to optimize the delicate trade-off between the primary RL objective and the ethical shaping rewards. GPT-4o-mini consistently achieved the most stable equilibrium across both tasks, validating its selection as the backbone for AMULED.

## 5 Discussion

We present two simple scenarios where an ethics-based shaping algorithm helps RL agents make ethically sound decisions while still achieving their primary objectives. These scenarios serve as placeholders for trivial daily activities, demonstrating how ethical considerations can be seamlessly integrated into routine tasks within reinforcement learning environments. Leveraging large language models to inform agent behavior allows us to build on existing work that highlights the limitations of traditional reward shaping. Our approach not only enhances ethical performance but also demonstrates the importance of incorporating diverse moral theories, addressing moral uncertainty effectively. By addressing ethical objectives often neglected in traditional reinforcement learning, our approach ensures that ethical considerations are integrated without compromising the primary objectives, paving the way for more holistic and responsible AI systems (Radanliev et al., 2024).

Our experiments weigh each of the moral clusters equally. Without loss of generality, we argue that our framework introduces the concept of choosing between ethical models as “moral clusters”. One can choose to prioritize certain clusters through weights (as each culture reflects different emphasis on different moral values). One can also look at the moral agents as feedback from different human agents, who may have different takes on ethical choices but in aggregation lead to a *best* solution. However, these approaches are beyond the scope of this work, whose primary aim is to introduce this paradigm. Furthermore, we emphasize that AMULED is intended as a practical and modular framework for integrating moral uncertainty into reinforcement learning, and does not provide formal guarantees such as policy convergence or strict safety bounds. While the approach builds on PPO, a method with known empirical stability properties, and incorporates a KL-divergence constraint to limit policy deviation, developing formal theoretical guarantees remains an important direction for future work.

From our simple examples, we see significant improvement in behavior when incorporating diverse moral philosophies, showing the importance of moral decision-making in RL contexts. Our ethics-shaping approach simplifies the design of a value-aligned RL system. This approach divides the complex task into two distinct layers: the first layer focuses on achieving the primary goal, while the second, ethical layer refines the outcome to meet the secondary ethical goal. This two-layered structure allows the system to address core objectives initially, followed by ethical adjustments to enhance overall responsibility. However, we do see that this approach is sensitive to i) the reasoning quality of the language model, and ii) the availability of feedback samples to shape learning. Language models do not have the long-term planning to guide their reasoning, which makes them underperform a well-crafted reward function for long-term, spatial tasks. In addition, LLM-based feedback may inherit biases present in their training data and can exhibit inconsistent reasoning across contexts, including known weaknesses in spatial and multi-step decision-making. These limitations directly affect the quality of the moral signals used for reward shaping. While AMULED mitigates this through multi-moral aggregation and a modular design that allows replacement or refinement of underlying models, it does not eliminate these issues entirely. Human samples on the other hand would provide the best “human-aligned” feedback but might be too sparse when dealing with large and complex state-action spaces. We also see that different moral frameworks can result in different priorities for performing the ethical tasks. These insights emphasize the value of understanding and optimizing moral frameworks for developing agents capable of addressing complex ethical challenges. Overall, our research tries to lay a solid foundation for future exploration into enhancing moral reward systems and improving spatial reasoning in large language models.

In the context of this study, we validate the ethical “correctness” of the agent's decisions by comparing them against the “Human” heuristic baselines shown in our results. These baselines were designed to encode widely accepted moral norms (such as minimizing harm to vulnerable agents) and serve as a proxy for ground truth. The observed alignment between AMULED's behavior and these heuristics demonstrates the model's capability to internalize intended ethical values. We acknowledge, however, that this is a technical validation; determining whether these decisions align with the nuanced, subjective judgment of real human observers is a critical next step that we reserve for future empirical research.

Our findings contribute significantly to the ongoing discourse in the intersection of AI and ethics, particularly within reinforcement learning frameworks. As innovation continues to drive autonomous technologies, ethical decision-making becomes increasingly crucial in autonomous systems. Although our work presents promise in using ethically aligned LLM agents to integrate moral reasoning into AI, it should be viewed as complementary to the human-in-the-loop philosophy and is best suited for safe application in routine, low-stakes activities (e.g., personal assistant robots, kitchen assistant robots). For complex, high-stakes decision-making tasks with significant societal and community impacts, further research and rigorous testing are essential. Our ethical LLM agents work best to bootstrap learning algorithms, in cases where we would lack fine-grained feedback from human oversight. By design, eventually the LLM feedback can be replaced by feedback from not just one, but multiple human evaluators. Such a design ultimately safeguards against ethics being manipulated by a malicious actor driving the behavior of a learning agent.

While our framework aims to comprehensively represent major ethical paradigms for AI decision-making, we acknowledge that categorizing ethical theories into distinct clusters may oversimplify the complexities and intersections among different moral philosophies. This structured approach is intended to facilitate the practical implementation of ethical reasoning in AI systems. However, operationalizing these theories involves abstracting intricate philosophical ideas while we strive to preserve the core principles of each ethical approach. We selected representative theories and decision factors based on their prominence and relevance in the literature, recognizing that some degree of subjectivity is inherent in this process.

We acknowledge that our use of fixed and separable ethical clusters represents a design choice that prioritizes clarity and tractability over capturing the full richness of moral reasoning. In practice, human decision-making rarely follows a single moral theory rigidly. Instead, individuals often blend elements from different ethical traditions depending on the context, and this hybrid reasoning may vary substantially across cultural or societal settings. For instance, care-oriented considerations may be weighted more heavily in collectivist societies, while deontological rules might dominate in legalistic contexts. In this paper we intentionally adopt separable clusters as a first step, as this provides a transparent mapping from well-established philosophical frameworks to computational representations, and makes belief aggregation methods easier to analyze. However, AMULED's modular structure is designed to be extendable: future work could allow agents to dynamically adjust cluster weights, incorporate hybrid reasoning patterns, or learn culturally specific moral priors from human feedback. This flexibility would enable the framework to better mirror the nuanced and context-dependent way that people actually reason about ethics, while retaining the interpretability benefits of our current design.

Our framework is based on key assumptions: we believe that the five clusters effectively encompass the major streams of ethical thought pertinent to AI decision-making and that the selected theories within each cluster are sufficiently representative of their respective ethical approaches. We also assume that the identified key concepts and decision factors can be meaningfully translated into computational models, providing a solid foundation for future research. AMULED demonstrates strong generalization across diverse tasks like the static “Finding Milk” (grid-based planning with immediate ethics) and dynamic “Driving and Rescuing” (continuous motion, probabilistic elements, high-stakes decisions), highlighting its adaptability to varying environmental complexities and moral conflicts. The framework's task-agnostic ethical layer, integrating RL-LLM for scalable feedback, few-shot prompting for precise outputs, and multi-moral belief aggregation reduces dependence on hand-crafted rewards and balances primary goals with ethical sub-goals without domain-specific adjustments. Evidence from results shows consistent ethical alignment and superior outcomes over baselines in both tasks, suggesting robustness to moral uncertainty and potential extension to other domains (e.g., healthcare triage, resource allocation) via simple prompt and environment tweaks, without core architecture redesign.

Recognizing that different countries and cultures can value certain moral beliefs over another, AMULED was designed to have modular selection of ethical frameworks, rather than imposing a single, moral philosophy to drive decisions. While we highlight that this modular, multi-moral approach avoids the pitfalls of sticking to a fixed moral framework, we do recognize that the representation of belief values generated from LLMs may be prone to external biases and subjectivity. For instance, models trained predominantly on Western data may implicitly favor individualistic interpretations of “justice” or “autonomy” over collectivist perspectives. AMULED mitigates this by enforcing distinct philosophical lenses, which prevents the system from defaulting to a single normative perspective. Moreover, our modular design allows specific clusters to be fine-tuned or swapped for culturally diverse alternatives (e.g., non-Western virtue ethics) without retraining the entire agent. This complicates the design of a universally accepted modular decision-making framework. Moreover, the dynamic nature of ethics poses a challenge, as ethical norms can evolve over time and vary across cultures, potentially rendering static models ineffective. Accountability is another critical issue (Helbing, 2021); ambiguity arises regarding who is responsible for the outcomes generated by AI, whether it be the developers, the organization deploying the AI, or the model itself. To build trust and accountability, enhancing the transparency of the AMULED model's decision-making processes is essential, potentially through techniques that provide explanations, visualizations, or justifications for its ethical reasoning. This approach would improve user confidence and ensure that the AI's ethical framework aligns with societal values and norms (Helbing and Ienca, 2024).

The integration of Belief Probability Assignment (BPA) into reinforcement learning offers a transformative approach to ethical decision-making in AI systems. By aggregating beliefs from multiple feedback sources, such as ethical LLMs and human evaluators, AI can effectively assess and balance competing moral frameworks. This capability is particularly valuable in contexts marked by normative uncertainty, where ethical models like utilitarianism and deontology may provide conflicting guidance. Through this aggregation, the AMULED system evaluates decisions with a nuanced understanding of diversity and confidence across ethical theories, enabling iterative refinement of strategies that align with widely accepted moral standards. Our framework explicitly accommodates multiple ethical perspectives, leveraging techniques such as Dempster-Shafer Theory (DST) to reconcile conflicts and generate coherent probabilistic outputs under uncertainty. While DST provides a mathematically principled way to combine beliefs, some subtleties of ethical tension may remain less transparent, highlighting opportunities for improving interpretability and accountability. Future work could focus on providing explainable justifications, tracking the influence of individual moral perspectives, and integrating contextual or culturally informed norms, thereby enhancing trust and practical applicability in dynamic, real-world environments, including multi-agent systems and human-AI collaborations.

While AMULED demonstrates strong performance in simulated environments, we see its greatest value as a methodological foundation for integrating pluralistic moral frameworks into RL. In this work, our focus was on establishing the technical feasibility and proof-of-concept behavior of the framework. Assessing how well AMULED aligns with human ethical reasoning is a natural next step rather than the primary goal of this paper. Looking ahead, we envision two complementary validation paths. One is user studies where participants evaluate AMULED-generated decisions in scenarios such as FindMilk and Driving and Rescuing, providing ratings of agreement and moral acceptability. Another is expert annotation, where ethicists or domain specialists assess a subset of AMULED's decisions for consistency with philosophical frameworks and the clarity of their justification. By outlining these protocols, we position AMULED as both a step toward and a tool for empirical investigation of moral alignment, ensuring that future work can build directly on the methodological advances we present here.

## 6 Conclusion and future works

Our work advances the integration of ethical reasoning into AI, particularly in reinforcement learning frameworks, by leveraging ethically aligned LLM agents. While effective for low-stakes applications like personal or kitchen assistant robots, our approach is best viewed as complementary to human oversight, especially in high-stakes scenarios requiring rigorous testing. The framework excels in bootstrapping learning algorithms where human feedback is scarce, with the potential to replace LLM feedback with evaluations from multiple human evaluators, safeguarding against malicious manipulation.

Applications include smart home assistants, where AMULED promotes ethical and sustainable choices, and social media platforms, where it fosters constructive, inclusive interactions. However, categorizing ethical theories into clusters may oversimplify moral complexities, and operationalizing these theories involves abstracting philosophical principles. Our framework assumes that the selected ethical clusters and decision factors are representative and computationally translatable, though cultural and contextual variations pose challenges.

Future research should focus on hybrid ethical models, real-time adaptation, and interpretability to enhance trust and relevance. Addressing biases, exploring multi-agent scenarios, and establishing evaluation benchmarks are critical for equitable and robust outcomes. By integrating Belief Probability Assignment (BPA) into reinforcement learning, AMULED enables nuanced ethical decision-making, balancing competing moral frameworks in dynamic environments. Leveraging the Dempster-Shafer Theory for robust belief aggregation and the Jensen-Shannon Belief Divergence for precise information fusion, AMULED effectively synthesizes diverse ethical perspectives while quantifying uncertainty and resolving conflicts. These advanced techniques ensure that the system can adaptively weigh and harmonize competing moral values, such as utilitarianism and deontology, with a mathematically grounded approach. This paves the way for responsible, adaptable AI systems that are not only aligned with societal values but also capable of navigating the complexities of real-world ethical dilemmas with confidence and transparency.

To translate these theoretical capabilities into real-world implementation, two key practical challenges must be addressed: computational latency and safety verification. Regarding scalability, we emphasize that AMULED is designed as a training-time framework. The high computational cost of querying the LLM and performing belief aggregation is incurred only during training, and does not affect deployment. The resulting agent is a standard reinforcement learning policy that has internalized ethical priors, and therefore does not require any LLM calls at inference time. This design makes real-time deployment feasible, as decision-making latency is equivalent to that of conventional RL agents. The resulting output is a standard, lightweight RL policy that has internalized these ethical priors, allowing it to execute under real-time constraints without requiring live LLM inference. Furthermore, for implementation in high-stakes domains like healthcare or autonomous driving, the transition would necessitate standard safety-critical machine learning workflows. This includes rigorous offline evaluation using historical datasets to verify that the agent's learned policy aligns with expert decisions. It also includes comparative validation against human decision-making by running equivalent ethical scenarios with human participants to assess alignment between the learned policy and human judgments. This is followed by constrained deployment, where the agent operates with limited autonomy under human oversight. These steps ensure that the ethical behaviors learned via AMULED are validated against domain-specific safety requirements prior to full-scale deployment.

## Data Availability

The original contributions presented in the study are included in the article/Supplementary material, further inquiries can be directed to the corresponding author.
