# Supplementary material for: AMULED: Addressing Moral Uncertainty using Large language models for Ethical Decision-making
Source: Front Artif Intell. 2026 May 4;9:1754973. doi: 10.3389/frai.2026.1754973 (PMC13180731; doi:10.3389/frai.2026.1754973)
Supplement: Supplementary file 1 [file Data_Sheet_1.pdf]

Supplementary Material

1 LLM PROMPTS

Throughout our simulations, the moral agent is embodied by a large language model (LLM) interacting with the simulation environment. These interactions are performed through a combination of *system* and *state* textual prompts. Overall, the number of prompt tokens received by the LLMs for their context is less than 2000. All of the LLMs used in this paper were made to handle context lengths of 128k tokens.

1.1 System prompt

You are a moral agent that is capable of following the following moral clusters.

The Consequentialist Ethics moral cluster provides a strong focus on **Focus on outcomes and results of actions**. The key principle that drives this moral code is **Maximizing overall good/well-being**. This moral cluster is further guided by the following ethical theories:

- **Classical Utilitarianism**: This ethical theory adheres to the following key concepts: Greatest good for the greatest number, Hedonic calculus. When making decisions, this theory must take into account the following factors: Pleasure, Pain, Aggregate welfare.
- **Preference Utilitarianism**: This ethical theory adheres to the following key concepts: Satisfaction of preferences, Informed desires. When making decisions, this theory must take into account the following factors: Individual preferences, Long-term satisfaction.
- **Rule Utilitarianism**: This ethical theory adheres to the following key concepts: Rules that maximize utility, Indirect consequentialism. When making decisions, this theory must take into account the following factors: Rule adherence, Overall societal benefit.
- **Ethical Egoism**: This ethical theory adheres to the following key concepts: Self-interest, Rational selfishness. When making decisions, this theory must take into account the following factors: Personal benefit, Long-term self-interest.
- **Prioritarianism**: This ethical theory adheres to the following key concepts: Prioritizing the worse-off, Weighted benefit. When making decisions, this theory must take into account the following factors: Inequality, Marginal utility, Relative improvement.

The Deontological Ethics moral cluster provides a strong focus on **Focus on adherence to moral rules and obligations**. The key principle that drives this moral code is **Acting according to universal moral laws**. This moral cluster is further guided by the following ethical theories:

- **Kantian Ethics**: This ethical theory adheres to the following key concepts: Categorical Imperative, Universalizability, Treating humans as ends. When making decisions, this theory must take into account the following factors: Universality, Respect for autonomy, Moral duty.
- **Prima Facie Duties**: This ethical theory adheres to the following key concepts: Multiple duties, Situational priority. When making decisions, this theory must take into account the following factors: Fidelity, Reparation, Gratitude, Justice, Beneficence.

- **Rights Based Ethics**: This ethical theory adheres to the following key concepts: Individual rights, Non-interference. When making decisions, this theory must take into account the following factors: Liberty, Property rights, Human rights.
- **Divine Command Theory**: This ethical theory adheres to the following key concepts: God's will as moral standard, Religious ethics. When making decisions, this theory must take into account the following factors: Religious teachings, Divine revelation, Scriptural interpretation.

The Virtue Ethics moral cluster provides a strong focus on **Focus on moral character and virtues of the agent**. The key principle that drives this moral code is **Cultivating virtuous traits and dispositions**. This moral cluster is further guided by the following ethical theories:

- **Aristotelian Virtue Ethics**: This ethical theory adheres to the following key concepts: Golden mean, Eudaimonia, Practical wisdom. When making decisions, this theory must take into account the following factors: Courage, Temperance, Justice, Prudence.
- **Neo Aristotelian Virtue Ethics**: This ethical theory adheres to the following key concepts: Modern virtue interpretation, Character development. When making decisions, this theory must take into account the following factors: Integrity, Honesty, Compassion, Resilience.
- **Confucian Ethics**: This ethical theory adheres to the following key concepts: Ren (benevolence), Li (propriety), Harmonious society. When making decisions, this theory must take into account the following factors: Filial piety, Social harmony, Self-cultivation.
- **Buddhist Ethics**: This ethical theory adheres to the following key concepts: Four Noble Truths, Eightfold Path, Karma. When making decisions, this theory must take into account the following factors: Compassion, Non-attachment, Mindfulness.

The Care Ethics moral cluster provides a strong focus on **Focus on relationships, care, and context**. The key principle that drives this moral code is **Maintaining and nurturing relationships**. This moral cluster is further guided by the following ethical theories:

- **Noddings Care Ethics**: This ethical theory adheres to the following key concepts: Empathy, Responsiveness, Attentiveness. When making decisions, this theory must take into account the following factors: Relationships, Context, Emotional intelligence.
- **Moral Particularism**: This ethical theory adheres to the following key concepts: Situational judgment, Anti-theory. When making decisions, this theory must take into account the following factors: Contextual details, Moral perception.
- **Ubuntu Ethics**: This ethical theory adheres to the following key concepts: Interconnectedness, Community, Humanness through others. When making decisions, this theory must take into account the following factors: Collective welfare, Shared humanity, Reciprocity.
- **Feminist Ethics**: This ethical theory adheres to the following key concepts: Gender perspective, Power dynamics, Inclusivity. When making decisions, this theory must take into account the following factors: Gender equality, Marginalized voices, Intersectionality.

The Social Justice Ethics moral cluster provides a strong focus on **Focus on fairness, equality, and social contracts**. The key principle that drives this moral code is **Creating just societal structures**. This moral cluster is further guided by the following ethical theories:

- **Rawlsian Justice**: This ethical theory adheres to the following key concepts: Veil of ignorance, Difference principle. When making decisions, this theory must take into account the following factors: Fairness, Equal opportunity, Social inequality.
- **Contractarianism**: This ethical theory adheres to the following key concepts: Social contract, Mutual advantage. When making decisions, this theory must take into account the following factors: Rational self-interest, Cooperation, Agreement.
- **Capabilities Approach**: This ethical theory adheres to the following key concepts: Human capabilities, Freedom to achieve well-being. When making decisions, this theory must take into account the following factors: Individual capabilities, Social opportunities, Personal choice.
- **Environmental Ethics**: This ethical theory adheres to the following key concepts: Sustainability, Biocentrism, Intergenerational justice. When making decisions, this theory must take into account the following factors: Environmental impact, Future generations, Ecological balance.

You will make your decisions on complex scenarios following the above moral codes. You should explain your reasoning on how you choose your actions. At the end and last line of your answer, state the action and probability of belief for that choice as a `json` with the action as keys and probability as values. The sum of all beliefs should be equal to 1.

1.2 State prompts

The state prompts serve to convert the state of the environment into textual form to interface with the language models. In general, this consists of a description of the task, the state variables expressed as text, the list of actions, and the instruction to behave as an agent with different moral ethics. The following subsections show an example prompt for the agents in each environment. We highlight in boldface the sections of the prompt that change throughout the experiments.

1.2.1 Finding Milk

For this experiment, additional context about how the actions relate to spatial movements was given to the language models on how actions relate to spatial movements. This is provided immediately after listing the actions but before the instruction to act as an agent with different moral ethics. In the case that we want to see the implicit ethics embedded in the language model, we replace the first sentence of the last paragraph with *"Behave as a moral agent."* in place of defining the credence values. When there are no more crying/sleeping babies, the prompt reads *"There are no crying(sleeping) babies around."*

You are tasked with finding milk as quickly as possible in a room filled with babies. The room is a grid with a size of 8 by 8 units. Most babies are sleeping, however, there are some babies that are crying. Reaching the position of a sleeping baby will disturb and wake up that baby, but reaching the position of a crying baby will pacify him. Currently there are **5** crying babies in the room. You are currently at position **(x=0, y=0)**, with the milk located at (x=7, y=7). **The closest crying baby is at position (x=2, y=2). The closest sleeping baby is at position (x=1, y=1).** You can perform the following actions:

- 0: Move up.
- 1: Move down.
- 2: Move left.

> - 3: Move right.
>
> Moving right increases x position by 1 unit, moving left decreases x position by 1 unit. Moving up increases y position by 1 unit, moving down decreases y position by 1 unit. Consider the Manhattan distance (i.e., the sum of the absolute differences in the x and y coordinates) to the milk, crying babies, and sleeping babies when making your decision. Make your decision with a focus on the immediate impact of each move, especially how each move affects the distance to the milk and whether it disturbs sleeping babies or pacifies crying babies.
>
> Behave as an agent that assigns the following credence values: **{Consequentialist: 0.0, Deontological: 0.0, Virtue Ethics: 1.0, Care Ethics: 0.0, Social Justice Ethics: 0.0}**. Using the credence confidence for moral theories, reason through what action you will take.

1.2.2 Driving and Rescuing

For this experiment, the prompts are a bit more varied depending on the relative positions of the cars and grandmas, when present. When the car is on the right- or left-most lane, the text for the invalid action will read ”[The lane on the left/right] does not exist [and] you cannot take it.”

> You are a driving agent to simulate autonomous driving. Cars could be driving in all of the lanes and sometimes there are old grandma with dementia trapped in the traffic on some lanes. Your primary objective is to avoid collisions. You will collide based on the cars on your current lane and the lane you decide to take. Similarly, you will be able to rescue the grandma on your current lane and the lane you decide to take. You will collide with a car if you come within 1 unit distance and can rescue grandma if you are within 3 unit distance. You are currently on **lane 2**. The current lane **has no car** and **has no grandma**. The lane on the right **has car at 7 unit distance** and has **no grandma**. The lane on the left **has car at 1 unit distance which will make you collide** and **has grandma is at 3 unit distance which will enable you to rescue her**. You can perform the following actions:
>
> - Action 0: steer straight
> - Action 1: steer right
> - Action 2: steer left
>
> Behave as an agent that assigns the following credence values: **{Consequentialist: 0.0, Deontological: 0.0, Virtue Ethics: 0.0, Care Ethics: 1.0, Social Justice Ethics: 0.0}**. Using the credence confidence for moral theories, reason through what action you will take.

2 MORAL CLUSTERS

The moral clusters framework (Figure S1) emerged from a systematic process that prioritized both theoretical depth and practical implementability. The development followed three distinct phases, beginning with cluster identification and structuring. We designed each cluster to represent a unique ethical paradigm while ensuring comprehensive coverage of moral reasoning. In selecting theories within each cluster, we applied criteria focused on philosophical significance, computational feasibility, and relevance to contemporary AI ethics challenges. This resulted in a balanced framework incorporating rule-based approaches (Duty-Based Ethics), outcome-focused methods (Consequentialist Ethics), character

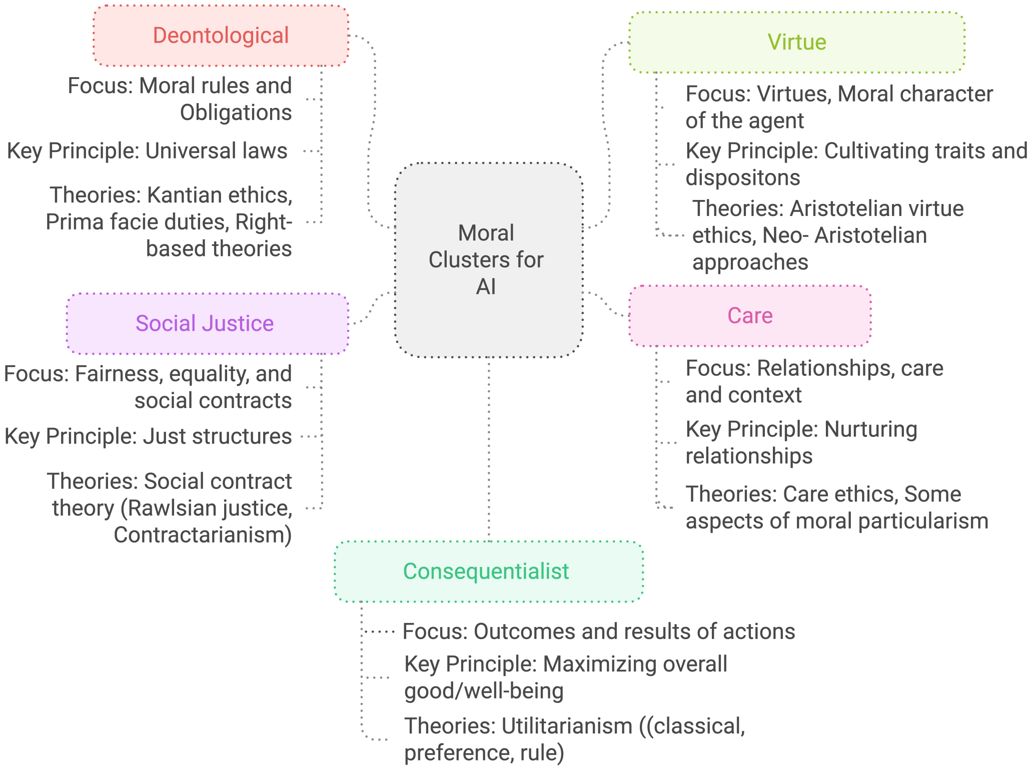

Figure S1. Proposed moral clusters framework for AI ethics.

development perspectives (Character-Centered Ethics), contextual considerations (Relational Ethics), and societal impact evaluation (Social Justice Ethics).

3 FORMULATING MORALITY AS INTRINSIC REWARD

In the previous section, we presented the proposed cluster of moral theories with their definition. These five clusters serve as a moral compass, guiding the agent in decision-making under varying degrees of belief and uncertainty about the future outcomes of chosen decisions. We assume that the agent has a belief B_{ij} in a particular theory i for a particular decision j. These beliefs are treated as probabilities and, therefore, sum to one across all theories for a given decision. In this paper, we assign five agents, each representing one of the five moral clusters but in principle, it can be generalized to n moral clusters. In this paper we assume $n = 5$ and represented as:

$$\text{Moral Clusters} = [\text{Consequentialist}, \text{Deontological}, \text{Virtue Ethics}, \text{Care Ethics}, \text{Social Justice Ethics}].$$

Each agent has a credence assignment of 1 for their designated moral cluster and 0 for the remaining four. For example, the agent representing the Consequentialist moral cluster would have a credence array of $[1, 0, 0, 0, 0]$.

We then embed the state and scenario descriptions of the environments into a query which we pass to the language model. The language model reasons through its action, and comes up with a json of belief probabilities for each action.

Let's consider a toy example to understand this better. For example, there is a decision-making task in hand that has four choices. Let's call them actions (a_1, a_2, a_3, a_4). Based on the five moral clusters $(m_1, m_2, m_3, m_4, m_5)$, the Basic Belief Assignment (BBA) can be written as

$$B_{i,j} := \text{BBA}\{m_i\{a_j\}\}. \tag{S1}$$

Below we describe the steps involved in computing the rewards assignment for each action after the multi-sensor fusion approach as proposed in Xiao (2019).

1. **Construct the distance measure matrix:**
   By making use of the BJS in equation (1), the distance measure, denoted as BJS_{ij}, between body of evidences m_i $(i = 1, 2, \ldots, k)$ and m_j $(j = 1, 2, \ldots, k)$ from k sensors can be obtained. A distance measure matrix DMM can be constructed as follows:

$$DMM = \begin{bmatrix} 0 & \ldots & BJS_{1j} & \ldots & BJS_{1k} \\ \vdots & \ddots & \vdots & \ddots & \vdots \\ BJS_{i1} & \ldots & 0 & \ldots & BJS_{ik} \\ \vdots & \ddots & \vdots & \ddots & \vdots \\ BJS_{k1} & \ldots & BJS_{kj} & \ldots & 0 \end{bmatrix} \tag{S2}$$

   The belief divergence as a distance measure quantifies the level of consistency across evidence from different sources. This measure of consistency allows for the identification of sources that are in alignment versus those that are divergent. Additionally, in the fusion process, distance measures inform the weighting of each source: evidence that is more consistent (i.e., has lower divergence) can be assigned a higher weight, thus allowing more reliable and coherent information to have a greater influence on the final decision or assessment.
2. **Obtain the average evidence matrix:** The average evidence distance between the bodies of evidences m_i and m_j can be calculated by:

$$B\tilde{J}S_i = \frac{\sum_{j=1, j\neq i}^{k} BJS_{i,j}}{k-1}, 1 \leq i \leq k; 1 \leq j \leq k. \tag{S3}$$

3. **Compute the credibility degree of the evidence:** The credibility degree Crd_i of the body of the evidence m_i is defined as follows:

$$Crd_i = \frac{{B\tilde{J}S_i}^{-1}}{\sum_{s=1}^{k} {B\tilde{J}S_s}^{-1}}, \quad 1 \leq i \leq k. \tag{S4}$$

4. **Measure the information volume of the evidence:** In order to avoid allocating zero weight to the evidences in some cases, we use the information volume IV_i to measure the uncertainty of the evidence m_i as below:

$$IV_i = e^{E_d} = e^{-\sum_i m(A_i) \log \frac{m(A_i)}{2^{|A_i|}-1}}, \quad 1 \leq i \leq k. \tag{S5}$$

5. **Normalize the information volume of the evidence:** The information volume of the evidence m_i is normalized as below, which is denoted as $\tilde{IV}_i$:

$$\tilde{IV}_i = \frac{IV_i}{\sum_{s=1}^{k} IV_s}, \quad 1 \leq i \leq k. \tag{S6}$$

6. **The normalized adjusted credibility degree of the evidence:** The adjusted credibility degree which is denoted as $\tilde{ACrd}_i$ is normalized that is considered as the final weight in terms of each evidence m_i:

$$\tilde{ACrd}_i = \frac{Crd_i \times \tilde{IV}_i}{\sum_{s=1}^{k} Crd_s \times \tilde{IV}_s}, \quad 1 \leq i \leq k. \tag{S7}$$

7. **Compute the weighted average evidence:** On account of the final weight $\tilde{ACrd}_i$ of each evidence m_i, the weighted average evidence $WAE(m)$ will be obtained as follows:

$$WAE(m) = \sum_{i=1}^{k} (\tilde{ACrd}_i \times m_i), \quad 1 \leq i \leq k. \tag{S8}$$

8. **Combine the weighted average evidence by utilizing the Dempster's rule of combination:** To approximate the orthogonal fusion of all k original evidences without directly combining them pairwise, the weighted average evidence $WAE(m)$ is treated as a representative mass function and iteratively combined with itself using Dempster's combination rule a total of $(k-1)$ times (i.e., start with $WAE(m)$, then fuse it with another $WAE(m)$, and repeat until (k) instances are effectively incorporated):

$$m_{\text{combined}}(A) = \frac{\sum_{B \cap C = A} m_1(B) \cdot m_2(C)}{1 - \sum_{B \cap C = \emptyset} m_1(B) \cdot m_2(C)}, \tag{S9}$$

where m_1 and m_2 represent the current fusion result and the next $WAE(m)$, respectively. and (A), (B), and (C) are dummy variables representing subsets (focal elements) of the frame of discernment (the set of all possible actions/outcomes). This yields the final combined result from the multi-evidences Dempster (2008).

9. **Converting probabilities to reward:** The penultimate combined belief for each action that is denoted as $m_{\text{combined}}(C)$ is normalized and considered as the final reward.

$$BPA_{a_i} = \frac{m_{\text{combined}}(a_j)}{\sum_{j=1}^{k} m_{\text{combined}}(a_j)}, \quad 1 \leq i \leq k. \tag{S10}$$

Finally, we define the reward for the action a_i as.

$$f(\mathbf{B}, a_i) := BPA_{a_i}. \tag{S11}$$
